# Expression and clinical significance of HSPA2 in pancreatic ductal adenocarcinoma

**DOI:** 10.1186/s13000-015-0253-9

**Published:** 2015-03-27

**Authors:** Hui Zhang, Hongli Gao, Chengli Liu, Yalin Kong, Cheng Wang, Hongyi Zhang

**Affiliations:** Department of Hepatobiliary Surgery, Air Force General Hospital of PLA, Beijing, 100142 China; Department of Oncology, the fourth people’s hospital of Jinan, Jinan, 250031 China

**Keywords:** HSPA2, Pancreatic cancer, Overall survival, Prognosis

## Abstract

**Background:**

It has been shown that heat shock-related 70-kDa protein 2 (HSPA2), a member of the HSP70 family of heat shock proteins, is important for cancer cell growth and metastasis. However, the status of HSPA2 expression and its prognostic significance in pancreatic cancer remain unknown.

**Methods:**

Quantitative reverse transcriptase ploymerase chain reaction (qRT-PCR) was applied to examine HSPA2 messenger RNA (mRNA) expression in 104 pairs of pancreatic cancer tissues and adjacent noncancerous tissues. Statistical analyses were applied to evaluate the diagnostic value and associations of HSPA2 expression with clinicopathological characteristics.

**Results:**

HSPA2 mRNA was significantly overexpressed in pancreatic cancer tissues (3.9 ± 0.8) than in adjacent normal tissues (1.1 ± 0.4) (P < 0.001). Clinicopathological analysis showed that HSPA2 expression was significantly correlated with tumor size (P = 0.024), histological differentiation (P = 0.012), TNM stage (P = 0.006), lymph node metastasis (P = 0.043) and serum CA19-9 level (P = 0.046). Moreover, patients with higher HSPA2 expression levels had shorter overall survival time than those with lower HSPA2 expression levels (P = 0.019). Furthermore, Cox regression analyses showed that HSPA2 expression was an independent predictor of overall survival (P = 0.011).

**Conclusions:**

Our results suggest that overexpression of HSPA2 in pancreatic cancer is associated with aggressive progression and poor prognosis and that HSPA2 may be served as a prognostic marker.

**Virtual slides:**

The virtual slide(s) for this article can be found here: http://www.diagnosticpathology.diagnomx.eu/vs/5988744821527257.

## Background

Pancreatic cancer remains to be one of the most challenging malignancies to treat. Surgical resection offers the only opportunity for cure. However, as no valid method for early detection of this disease has been established, 80% or more of patients present with unresectable disease at the time of diagnosis [1]. Furthermore, even when resection is performed, the recurrence rate is extremely high, resulting in the 5-year survival rate of patients with resected pancreatic cancer being no more than 20% [2]. Currently, carbohydrate antigen 19–9 (CA19-9) is commonly used for pancreatic cancer detection. However, the sensitivity and specificity of CA19-9 for the early diagnosis of pancreatic cancer are low [3]. Therefore, more accurate and acceptable tumor markers for the early detection of pancreatic cancer are needed.

Heat shock-related 70-kDa protein 2 (HSPA2, also known as HSP70-2) is a member of the HSP70 family of heat shock proteins [4]. The HSPA2 gene was originally characterized as the human counterpart of rodent genes which are specifically and highly expressed in the testis [5,6]. Recently, HSPA2 has attracted increased interest due to its possible involvement in carcinogenesis of non-testicular tissues. The overexpression of HSPA2 has been identified in several human malignancies, including non-small cell lung cancer [7], cervical carcinoma [8], esophageal squamous cell carcinoma [9], and hepatocellular carcinoma [10]. However, little is known about the expression and clinical significance of HSPA2 in pancreatic cancer. In this study, we therefore assessed the messenger RNA (mRNA) expression of HSPA2 in a series of pancreatic cancer specimens and investigated its associations with clinicopathological parameters and overall survival in patients with pancreatic cancer.

## Methods

### Patients and tissue specimens

A total of 104 consecutive patients with pancreatic ductal adenocarcinoma who underwent Whipple procedure at Air Force General Hospital of PLA between January 2009 and December 2012 were retrospectively reviewed. None of the patients had received chemotherapy or radiotherapy before surgery. Fresh tissues including pancreatic cancer tissues and adjacent normal tissues were collected and immediately snap-frozen in liquid nitrogen after surgery and were stored at −196°C until used. Patient preoperative demographic and clinical data, including age, gender, details of pathological diagnosis, serum CA 19–9 levels, follow-up period, and overall survival were collected prospectively. Patients were given postoperative adjuvant chemotherapy every four weeks for three months (Gemcitabine 1000 mg/m^2^ on days 1, 8, and 15). The study has been conducted in accordance with the ethical standards and the principles of the Declaration of Helsinki and has been approved by the Institutional Review Board of Air Force General Hospital of PLA. Written informed consent was obtained from all of the patients.

### qRT-PCR

Quantitative reverse transcriptase polymerase chain reaction (qRT-PCR) was utilized to detect HSPA2 expression in pancreatic cancer tissues. Briefly, total RNA was extracted using TRIzol extraction liquid (Invitrogen, Carlsbad, CA, USA) according to the manufacturer’s instructions. β-actin was used as an internal control. The reverse transcriptase (RT) reaction contained 10 ng of total RNAs, 50 nmol/l stem-loop RT primer, 1 × RT buffer, 0.25 mmol/l each of deoxynucleotide triphosphate (dNTP), 3.33U/μl MultiScribe reverse transcriptase, and 0.25U/μl RNase Inhibitor. The 20 μl reaction volumes were incubated at 16°C for 30 min, 40°C for 30 min, and 85°C for 5 min. Real-time PCR was then performed on a StepOnePlus real-time PCR system (Applied Biosystems, Foster City, CA, USA). The sequences of the primers were as follows: human HSPA2 forward 5’-TTCCACTCAGGCCGCGTCCG-3’ and reverse 5’-AATCGGGCCTTGGCAATCGTT-3’ and human β-actin forward 5’-CAAGAGATGGCCACGGCTGCT-3’ and reverse 5’-TCCTTCTGCATCTGTCGGCA-3’. The following PCR parameters were used: 95°C for 2 min, followed by 35 cycles of 95°C for 30 sec and 60°C for 30 sec and a final elongation step of 72°C for 10 min. All reactions were performed in triplicate and the cycle threshold (CT) value in each reaction well was recorded. The relative quantification of HSPA2 mRNA expression was calculated using the 2^-∆∆CT^ method.

### Statistical analysis

The HSPA2 mRNA expression level was expressed as mean ± standard deviation (SD). Associations between HSPA2 mRNA expression level in pancreatic cancer and clinicopathological features were determined using the *χ*^2^-test. The Kaplan-Meier method was used to estimate survival rates, and the log-rank test was used to assess survival differences between groups. The Cox proportional hazards model for multivariate survival analysis was used to assess predictors related to overall survival. All statistical analyses were performed using SPSS software (SPSS 19.0, Chicago, IL, USA) and P < 0.05 was considered statistically significant.

## Results

### HSPA2 mRNA upregulation in pancreatic cancer

The clinicopathological features of all the patients were summarized in Table 1. The patients were aged from 41 to 86 years with a median of 62 years. The degree of differentiation was well differentiated in 13 cases, moderately differentiated in 43 cases, and poorly differentiated in 48 cases. A total of 29 cases had lymph node metastases, while the remaining 75 cases did not have lymph node metastases. The clinical TNM stage according to the 7^th^ edition of the AJCC cancer staging manual was 27 cases of stage IA, 39 cases of stage IB, 28 cases of stage IIA, and 10 cases of stage IIB. Serum CA19-9 and bilirubin were elevated in 77 and 69 patients, respectively.

**Table 1:** **Association between HSPA2 mRNA expression and clinicopathological features of pancreatic cancer**

**Variables**	**No. of cases (n = 104)**	**HSPA2 mRNA expression**		***P*** **-value**
		**Low (n, %)**	**High (n, %)**	
Age (years)				0.484
<60	43	21 (48.8)	22 (51.2)	
≥60	61	28 (44.3)	33 (54.1)	
Gender				0.396
Male	82	37 (45.1)	45 (54.9)	
Female	22	12 (54.5)	10 (45.5)	
Tumor size (cm)				0.024
<2	47	32 (68.1)	15 (31.9)	
≥2	57	17 (29.8)	40 (70.2)	
Histological grades				0.012
Well/Moderate	56	32 (57.0)	24 (43.0)	
Poor	48	17 (35.4)	31 (64.6)	
TNM stage^a^				0.006
IA-IB	66	35 (53.0)	31 (47.0)	
IIA-IIB	38	14 (36.8)	24 (63.2)	
Lymph node metastasis				0.043
No	75	39 (48.0)	36 (52.0)	
Yes	29	10 (44.8)	19 (55.2)	
Vascular invasion				0.062
No	80	39 (48.8)	41 (51.2)	
Yes	24	10 (41.7)	14 (58.3)	
CA19-9 (U/ml)				0.046
<27	27	14 (51.9)	13 (48.1)	
≥27	77	35 (45.5)	42 (54.5)	
Bilirubin (μmol/l)				0.178
<17.1	35	16 (45.7)	19 (54.3)	
≥17.1	69	33 (47.8)	36 (52.3)	

^a^TNM staging was classified according to the 7^th^ edition of the AJCC cancer staging manual.

*HSPA2*, Heat shock-related 70 kDa protein 2.

**Figure 1:**
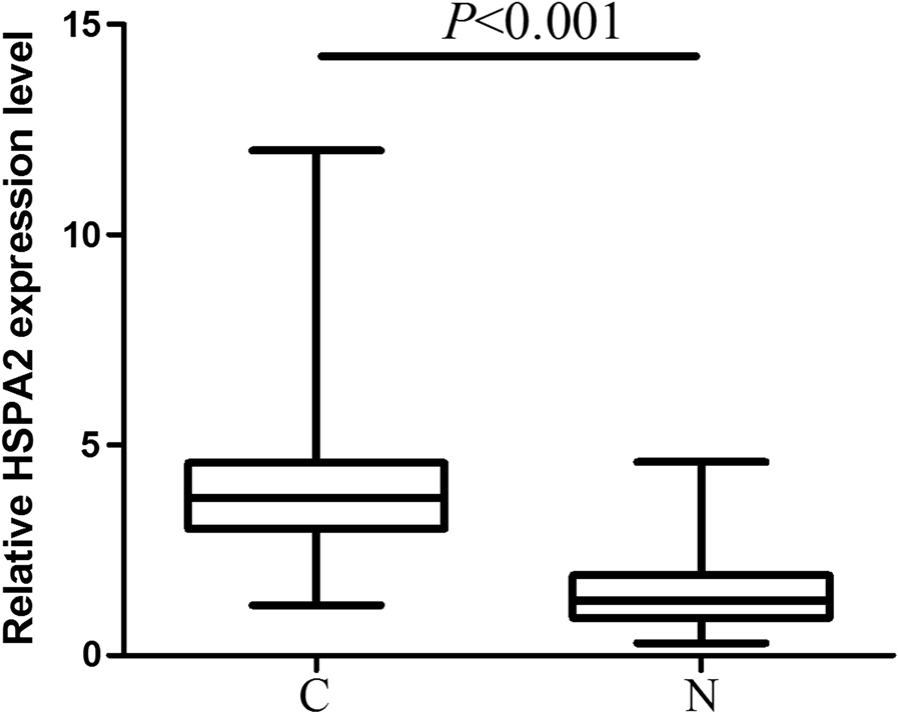
**Relative expression of HSPA2 in 104 pairs of pancreatic cancer tissues (C) and adjacent normal tissues (N).**

HSPA2 mRNA expression was detected in 104 pairs of pancreatic cancer and adjacent normal tissues by real-time quantitative RT-PCR. As shown in Figure 1, after normalization to β-actin expression levels, the expression level of HSPA2 in pancreatic cancer tissues (3.9 ± 0.8) was significantly higher than that in adjacent normal tissues (1.1 ± 0.4) (P < 0.001). The median HSPA2 mRNA expression level of pancreatic cancer tissues (4.1) was used as a cut-off point to divide all 104 patients into two groups: pancreatic cancer patients who expressed HSPA2 at levels less than the cut-off value were assigned to the low expression group (n = 49), and those with HSPA2 mRNA expression higher than the cut-off value were assigned to the high expression group (n = 55).

### Association between HSPA2 mRNA upregulation and clinicopathological parameters of patients with pancreatic cancer

Table 1 summarized the association between HSPA2 mRNA expression and clinicopathological parameters in pancreatic cancer. HSPA2 mRNA expression levels were significantly higher in the cancerous tissues of patients with IIA-IIB stage pancreatic cancer than those with IA-IB stage (P = 0.006). In addition, HSPA2 mRNA was expressed at significantly higher levels in patients with larger tumor sizes (P = 0.024). Moreover, we found that poorly differentiated tumors expressed higher HSPA2 than well or moderately differentiated tumors (P = 0.012). Finally, there were sufficient evidence to confirm its correlation with lymph node metastasis and serum CA19-9 level in pancreatic cancer (P = 0.043 and P = 0.046, respectively).

### Association between HSPA2 mRNA upregulation and poor prognosis in patients with pancreatic cancer

**Figure 2:**
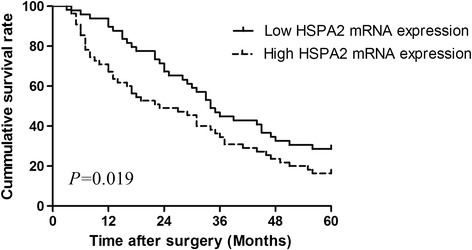
**Kaplan-Meier survival analysis for high HSPA2 mRNA expression level versus low HSPA2 mRNA expression level in patients with pancreatic cancer.**

The association between HSPA2 mRNA expression and overall survival of pancreatic cancer patients was investigated by Kaplan-Meier analysis and log-rank test. During the follow-up period, 81 of the 104 patients (77.9%) died. As shown in Figure 2, pancreatic cancer patients with low HSPA2 mRNA expression level had longer overall survival time than those with high HSPA2 mRNA expression level (log-rank test: P =0.019). Univariate analysis demonstrated that TNM stage (P = 0.013), the status of lymph node metastasis (P = 0.040) and vascular invasion (P = 0.017), and HSPA2 mRNA expression level (P = 0.007) were significantly associated with overall survival of pancreatic cancer patients (Table 2). No significant associations with patient survival were found among age at diagnosis, gender, tumor size, histological grade, serum CA19-9 level, and bilirubin. Multivariate analysis using the Cox proportional hazards model for all variables that were significant in the univariate analysis showed that TNM stage (P = 0.026), the status of vascular invasion (P = 0.042), and HSPA2 mRNA expression level (P = 0.011) were independent prognostic factors for overall survival in patients with pancreatic cancer (Table 2).

**Table 2:** **Univariate and multivariate analyses of prognostic factors in patients with pancreatic cancer**

**Variables**	**Univariable** ***P*** **-value (log-rank test)**	**Multivariable**	
		**HR (95% CI)**	***P*** **-value**
Age at diagnosis (years)			
<60 vs. ≥60	0.584		
Gender			
Male vs. Female	0.763		
Tumor size (cm)			
<2 vs. ≥2	0.082		
Histological grades			
Well/Moderate vs. Poor	0.067		
TNM stage^a^			
IA-IB vs. IIA-IIB	0.013	1.23 (1.14-1.52)	0.026
Lymph node metastasis			
No vs. Yes	0.040	1.14 (0.97-1.41)	0.054
Vascular invasion			
No vs. Yes	0.017	1.27 (1.04-1.49)	0.042
CA19-9 (U/ml)			
<27 vs. ≥27	0.082		
Bilirubin (μmol/l)			
<17.1 vs. ≥17.1	0.185		
HSPA2 mRNA expression			
Low vs. High	0.007	1.58 (1.11-1.89)	0.011

^a^TNM staging was classified according to the 7^th^ edition of the AJCC cancer staging manual.

*HSPA2*, Heat shock-related 70 kDa protein 2.

## Discussion

HSPA2, as a testis-specific protein, played an important role in spermatogenesis [5,6]. Recently, research has shown that human tumor cells can express HSPA2 at high levels [11,12]. The polymorphism of HSPA2 at position 1267 has been suggested to be associated with carcinogenesis in several malignant cancer tissues, such as lung cancer, cervical cancer, esophageal squamous cell carcinoma, and hepatocellular carcinoma [7-10]. Previous study has shown that HSPA2 was not found in normal pancreas [13]. However, whether HSPA2 is highly expressed in pancreatic cancer is unclear. For a more comprehensive insight into the clinical value of HSPA2 in pancreatic cancer, we performed a real-time quantitative RT-PCR assay to explore the mRNA expression level of HSPA2 as well as to investigate its association with clinicopathological features of pancreatic cancer patients. Our data revealed that the mRNA expression level of HSPA2 was significantly upregulated in pancreatic ductal adenocarcinoma and its expression was correlated with tumor size, tumor differentiation, TNM stage, lymph node metastasis, and serum CA19-9 levels. More importantly, we also demonstrated that the mRNA expression of HSPA2 was an independent prognostic predictor for overall survival.

Ductal adenocarcinoma is the most common type of pancreatic cancer, accounting for over 85% of cases [14]. It is the fourth leading cause of cancer death in the United States with a median survival of less than 6 month and a dismal 5-year survival rate of 3%-5% [15]. The cancer’s lethal nature stems from its propensity to rapidly disseminate to the lymphatic system and distant organs [16]. This aggressive biology and resistance to conventional and targeted therapeutic agents leads to a typical clinical presentation of incurable disease at the time of diagnosis. In the current study, we found that HSPA2 expression was proved to be associated with tumor size, histologic grade, tumor stage, and lymph node metastasis, strongly suggesting that HSPA2 might be involved in the carcinogenesis, development, progression, and metastasis of pancreatic ductal adenocarcinoma. More importantly, we proved that HSPA2 mRNA expression was significantly associated with overall survival of patients with pancreatic cancer. In support of this, Kaplan-Meier analysis of overall survival showed that patients whose tumors had higher HSPA2 expression tend to have a significantly worse overall survival, indicating that a high HSPA2 level is a marker of poor prognosis for patients with pancreatic cancer. Moreover, the Cox proportional hazards model showed that HSPA2 was a marker of poor overall survival independent of the known clinical prognostic indicators such as TNM stage and vascular invasion. Therefore, it could constitute a molecular prognostic marker for these patients, identifying who are more likely to have higher risk of death; thus, good candidates are to receive more aggressive treatments.

## Conclusions

In summary, we have reported the differential expression of HSPA2 in pancreatic cancer and adjacent normal tissues. Our results suggest that high expression of HSPA2 in pancreatic cancer is associated with poor overall survival and that HSPA2 may be served as a prognostic marker for pancreatic cancer.

## Abbreviations

CA19-9: Carbohydrate antigen 19–9
CT: Cycle threshold
dNTP: Deoxynucleotide triphosphate
HSPA2: Heat shock-related 70-kDa protein 2
mRNA: messenger RNA
qRT-PCR: Quantitative reverse-transcription polymerase chain reaction
RT: Reverse transcriptase
SD: standard deviation

## References

[1] Siegel R, Naishadham D, Jemal A (2012). Cancer statistics, 2012. CA Cancer J Clin.

[2] Liao WC, Chien KL, Lin YL, Wu MS, Lin JT, Wang HP (2013). Adjuvant treatments for resected pancreatic adenocarcinoma: a systematic review and network meta-analysis. Lancet Oncol.

[3] Jin XL, Xu B, Wu YL (2014). Detection of pancreatic cancer with normal carbohydrate antigen 19–9 using protein chip technology. World J Gastroenterol.

[4] Bonnycastle LL, Yu CE, Hunt CR, Trask BJ, Clancy KP, Weber JL (1994). Cloning, sequencing, and mapping of the human chromosome 14 heat shock protein gene (HSPA2). Genomics.

[5] Dix DJ, Allen JW, Collins BW, Poorman-Allen P, Mori C, Blizard DR (1997). HSP70-2 is required for desynapsis of synaptonemal complexes during meiotic prophase in juvenile and adult mouse spermatocytes. Development.

[6] Dix DJ, Allen JW, Collins BW, Mori C, Nakamura N, Poorman-Allen P (1996). Targeted gene disruption of Hsp70-2 results in failed meiosis, germ cell apoptosis, and male infertility. Proc Natl Acad Sci U S A.

[7] Scieglinska D, Gogler-Piglowska A, Butkiewicz D, Chekan M, Malusecka E, Harasim J (2014). HSPA2 is expressed in human tumors and correlates with clinical features in non-small cell lung carcinoma patients. Anticancer Res.

[8] Garg M, Kanojia D, Saini S, Suri S, Gupta A, Surolia A (2010). Germ cell-specific heat shock protein 70–2 is expressed in cervical carcinoma and is involved in the growth, migration, and invasion of cervical cells. Cancer.

[9] Zhang H, Chen W, Duan CJ, Zhang CF (2013). Overexpression of HSPA2 is correlated with poor prognosis in esophageal squamous cell carcinoma. World J Surg Oncol.

[10] Fu Y, Zhao H, Li XS, Kang HR, Ma JX, Yao FF (2014). Expression of HSPA2 in human hepatocellular carcinoma and its clinical significance. Tumour Biol.

[11] Rohde M, Daugaard M, Jensen MH, Helin K, Nylandsted J, Jaattela M (2005). Members of the heat-shock protein 70 family promote cancer cell growth by distinct mechanisms. Genes Dev.

[12] Scieglinska D, Piglowski W, Mazurek A, Malusecka E, Zebracka J, Filipczak P (2008). The HspA2 protein localizes in nucleoli and centrosomes of heat shocked cancer cells. J Cell Biochem.

[13] Scieglinska D, Piglowski W, Chekan M, Mazurek A, Krawczyk Z (2011). Differential expression of HSPA1 and HSPA2 proteins in human tissues; tissue microarray-based immunohistochemical study. Histochem Cell Biol.

[14] Ottenhof NA, de Wilde RF, Maitra A, Hruban RH, Offerhaus GJ (2011). Molecular characteristics of pancreatic ductal adenocarcinoma. Patholog Res Int.

[15] Hezel AF, Kimmelman AC, Stanger BZ, Bardeesy N, Depinho RA (2006). Genetics and biology of pancreatic ductal adenocarcinoma. Genes Dev.

[16] La Torre M, Nigri G, Petrucciani N, Cavallini M, Aurello P, Cosenza G (2014). Prognostic assessment of different lymph node staging methods for pancreatic cancer with R0 resection: pN staging, lymph node ratio, log odds of positive lymph nodes. Pancreatology.

